# Nitroalkene inhibition of pro-inflammatory macrophage effector function via modulation of signaling metabolite levels

**DOI:** 10.3389/fphys.2025.1426102

**Published:** 2025-10-16

**Authors:** Emily R. Stevenson, James P. O’Brien, Allison M. Manuel, Crystal E. Uvalle, Gregory J. Buchan, Steven J. Mullett, Karina Lockwood, Tomeka Suber, Bruce A. Freeman, Stacy L. Gelhaus

**Affiliations:** ^1^ Department of Pharmacology and Chemical Biology, University of Pittsburgh School of Medicine, Pittsburgh, PA, United States; ^2^ Health Sciences Mass Spectrometry Core, University of Pittsburgh, Pittsburgh, PA, United States; ^3^ Department of Pulmonary, Allergy, Critical Care and Sleep Medicine, University of Pittsburgh, Pittsburgh, PA, United States

**Keywords:** metabolism, inflammation, nitroalkene fatty acid, glutamine metabolism, macrophage

## Abstract

**Introduction:**

Classically activated innate immune cells undergo a metabolic switch to aerobic glycolysis to support effector function. We report that the small-molecule nitroalkene 10-n-octadec-9-enoic acid (NO_2_-OA) attenuates the Warburg- like phenotype of aerobic glycolysis in lipopolysaccharide (LPS)-activated macrophages, thus inhibiting pro-inflammatory signaling.

**Methods:**

RAW264.7 and bone marrow derived macrophage were treated with LPS with and without NO_2_-OA or 1400W. Pro-inflammatory cytokines were measured by ELISA and protein expression was determined by immunoblot. Central carbon metabolites with and without ^13^C stable isotope tracing were measured using liquid chromatography-high resolution mass spectrometry.

**Results:**

Overall, the present observations indicate that nitroalkene-induced changes in central carbon metabolism contribute to the anti-inflammatory actions of this class of multi-target lipid signaling mediators. Comparison of macrophage responses to NO_2_-OA with the inducible nitric oxide synthase (NOS2 and iNOS) inhibitor 1400W affirms that NO_2_-OA inhibition of NOS_2_ expression and activity alone was not sufficient to account for the decreases in pro-inflammatory cytokine expression. NO_2_-OA treatment reduced intracellular succinate levels, which may be attributed to a concomitant reduction in intracellular itaconate and reliance on glutamine, thereby contributing to hypoxia-inducible factor 1α (HIF1α) destabilization observed in LPS-activated macrophages.

**Conclusion:**

The current data provide additional perspective on the actions of this small-molecule electrophile, which is currently in a Phase 2 clinical trial for the treatment of obesity-related chronic pulmonary inflammation and associated airway dysfunction.

## Introduction

The pathogen-associated molecular pattern (PAMP)-mediated recognition receptor activation of toll-like receptors (TLRs) shifts innate immune cell metabolism from oxidative phosphorylation to aerobic glycolysis. PAMPs, such as lipopolysaccharide (LPS), signal through TLR-4 to activate nuclear factor kappa B (NF-κB)-regulated gene expression. From a metabolic perspective, this upregulates pro-inflammatory enzymes, including inducible nitric oxide synthase (NOS2), to produce nitric oxide (NO) (Mills et al., 2016; Palsson-McDermott et al., 2015). The high levels of NO generated by NOS2 can induce the nitration of unsaturated fatty acids (Vitturi et al., 2015), inhibit mitochondrial oxidative respiration via the nitrosation of complex I constituents, including nicotinamide adenine dinucleotide (NAD)H-binding catalytic module components, and promote competitive inhibition of complex IV (Chouchani et al., 2013; Clementi et al., 1998; Bailey et al., 2019). Decreasing oxidative phosphorylation, in turn, activates glycolytic enzyme expression and glycolytic flux to generate energy and increase lactate production.

The shift to enhanced glycolysis and *de novo* lipogenesis following exposure to an inflammatory stimulus enables cells to build biomass via anabolic reactions and meet energy demands. Metabolic changes in activated macrophages, such as the accumulation of the tricarboxylic acid (TCA) cycle intermediate succinate, are directly linked to macrophage inflammatory effector function (Mills et al., 2016; Harber et al., 2020; Tannahill et al., 2013). The increase in succinate levels is mediated by the upregulation of cis-aconitate decarboxylase (ACOD1/*Irg1*) activity, which catalyzes the production of itaconate. In turn, elevated itaconate concentrations competitively block the active site of succinate dehydrogenase (SDH), resulting in the accumulation of succinate and stabilization of hypoxia-inducible factor 1α (HIF1α) (Tannahill et al., 2013).

Natural and non-natural electrophilic nitroalkenes, along with other clinically utilized small-molecule electrophiles, inhibit NF-κB-directed pro-inflammatory cytokine and enzyme expression via the post-translational modification of functionally critical protein thiols in p50, p65, and at other points along the signaling cascade. This includes (a) inhibition of the inhibitor of NF-κB subunit kinase β (Iκκβ) phosphorylation and downstream inhibitor of NF-κB degradation, (b) alkylation of the NF-κB RelA p65 protein to prevent DNA binding, and (c) promotion of RelA polyubiquitination and proteasomal degradation (Cui et al., 2006; Villacorta et al., 2013; Wang et al., 2014; Wang et al., 2016; Khoo et al., 2018; Woodcock et al., 2018). Nitroalkenes are formed endogenously through metabolic and inflammation-induced reactions of NO and nitrite (NO_2_^−^), generating interest in further investigation of the mechanisms underlying endogenous signaling responses and their therapeutic potential in limiting pathogenic inflammatory processes (Schopfer et al., 2010; Klinke et al., 2014; Khoo et al., 2019; Zhou et al., 2021; Wilkinson et al., 2020). Nitroalkenes are reversibly reactive soft electrophiles that target highly conserved and kinetically susceptible members of the cysteine (Cys) proteome (Jobbagy et al., 2019; Nadtochiy et al., 2012; Turell et al., 2017). Nitroalkenes such as nitro-oleic acid (NO_2_-OA), in addition to attenuating inflammatory responses (Cui et al., 2006), also activate nuclear factor-erythroid 2-related factor 2 (Nrf2)-regulated repair and antioxidant enzyme expression (Villacorta et al., 2007). Of relevance to the data reported in this study, nitroalkenes reduce pro-inflammatory cytokine secretion by macrophages, neutrophils, T lymphocytes, vascular endothelial cells, and other cell types (Cui et al., 2006; Hwang et al., 2009; Choi et al., 2022). Moreover, the inhibition of NF-κB signaling suppresses NOS2 expression, which subsequently limits NO production and thus may restore oxidative phosphorylation in activated macrophages treated with NO_2_-OA.

In this study, we extend these insights to show that NO_2_-OA not only downregulates inflammatory signaling responses but also promotes inflammation resolution through alterations in TCA cycle metabolism that result in decreased concentrations of pro-inflammatory organic acids and increased synthesis of antioxidant response species. Our results reveal that the immunomodulatory activity of NO_2_-OA can be attributed to the suppression of both LPS-induced itaconate and succinate accumulation, along with a diminished metabolic reliance on extracellular glutamine resulting from the reappropriation of glutamine into glutathione (GSH) synthesis by NO_2_-OA. Notably, the inhibition of NOS2 by 1400W did not recapitulate NO_2_-OA-induced changes in cytokine production and central carbon metabolism, providing further evidence that NO_2_-OA mediates multiple signaling pathways to promote inflammation resolution. Finally, ACOD1/*Irg1* levels are lower in LPS-stimulated macrophages treated with NO_2_-OA, thus identifying a new mechanism of action of NO_2_-OA—the reduction of endogenous itaconate levels in inflammatory macrophages.

## Results

### Pharmacological inhibition of NOS2 by NO_2_-OA and 1400W inhibits NO production and pro-inflammatory cytokine secretion

**FIGURE 1:**
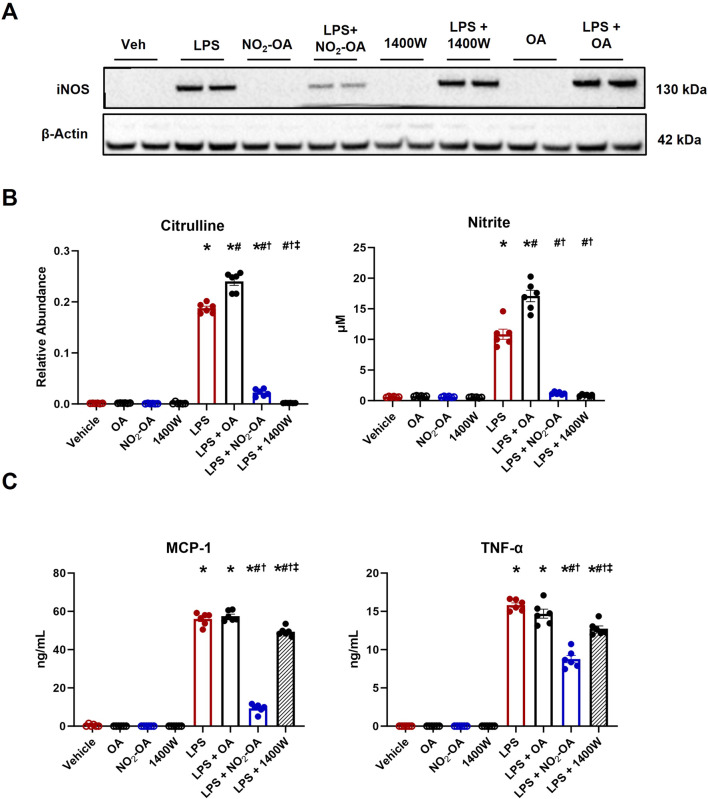
Pharmacological inhibition of NOS2 by NO_2_-OA and 1400W results in the inhibition of NO production and pro-inflammatory cytokine secretion. Wild-type RAW 264.7 macrophages treated with vehicle (0.01% DMSO), NO_2_-OA (5 µM), or 1400W (100 µM) were stimulated with LPS (10 ng/mL). Oleic acid (OA, 5 µM), the non-electrophilic fatty acid precursor of NO_2_-OA, was used as a negative control. Culture media and lysates were collected 12 h post-treatment. **(A)** Immunoblot analysis was performed for NOS2 on RAW 264.7 macrophage lysates. β-Actin (housekeeping control) is shown. Original blot was probed for both NOS2 and ACOD1/*Irg1* (Figure 2C; Supplementary Figure 1) such that the β-actin control is identical in both figures. **(B)** NOS2 by-products citrulline and nitrite were measured in cell lysates (citrulline) and media (nitrite) using liquid chromatography–high-resolution mass spectrometry and a UV spectrophotometer, respectively. **(C)** MCP-1 and TNF-α were measured through ELISA. *p* < 0.05 when compared to respective control (*), LPS (#), LPS + OA (†), and LPS + NO_2_-OA (‡) using ANOVA. Data are from n = 2 independent biological experiments, each comprising three technical replicates.

The shift from oxidative phosphorylation to glycolysis following LPS activation is considered a direct consequence of TLR4-induced NOS2 expression and increased production of NO, a key player in the metabolic reprogramming in innate immune cells (Bailey et al., 2019). We compared the metabolic responses of NOS2 inhibition via both NO_2_-OA and the selective NOS2 inhibitor 1400W. To investigate whether NO_2_-OA and 1400W similarly reduced pro-inflammatory metabolic signaling mediator responses, RAW 264.7 macrophages treated with 5 µM NO_2_-OA, 100 µM 1400W, or 0.01% DMSO (vehicle) were activated with 10 ng/mL LPS. Oleic acid (OA, 5 µM), the non-electrophilic fatty acid precursor of NO_2_-OA, was used as a negative control. First, it was confirmed that LPS induced NOS2 expression at 12 h. NO_2_-OA inhibited LPS-induced NOS2 expression, whereas 1400W and OA did not (Figure 1A; Supplementary Figure 1). Using liquid chromatography–high-resolution mass spectrometry (LC–HRMS), we measured relative amounts of citrulline, the other product of arginine metabolism. There was an increase in citrulline production in LPS-activated macrophages. Citrulline production decreased in macrophages treated with NO_2_-OA and 1400W, but not with OA (Figure 1B). Nitrite, a stable metabolite of NO oxidation, was measured in cell media using the Griess reaction and was observed to be increased in LPS-activated macrophages. Macrophage NO_2_^−^ generation was inhibited by both NO_2_-OA and 1400W, but not by non-electrophilic OA (Figure 1B). Finally, biomarkers of activation and inflammatory status, including MCP-1 and TNF-α, were measured through ELISA in cell supernatants at 12 h. LPS activation of macrophages increased the cytokine concentrations compared to controls, and both NO_2_-OA and 1400W decreased MCP-1 and TNF-α concentrations compared to OA (Figure 1C). In summary, LPS-activated RAW 264.7 macrophages treated with NO_2_-OA and 1400W inhibited NO production and downstream pro-inflammatory cytokine production.

### Pharmacological inhibition of NOS2 by 1400W does not recapitulate NO_2_-OA-induced changes in metabolism

**FIGURE 2:**
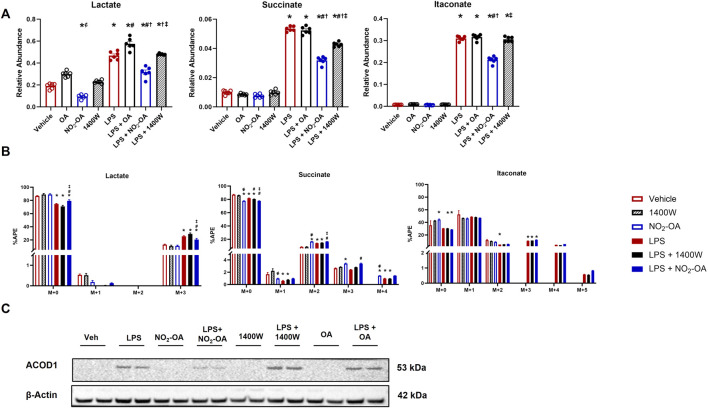
Pharmacological inhibition of NOS2 by 1400W does not recapitulate NO_2_-OA-induced changes in metabolism. Wild-type RAW 264.7 macrophages were treated with vehicle (0.01% DMSO, control), oleic acid (OA, 5 μM, control), NO_2_-OA (5 µM), or 1400W (100 µM), with and without LPS activation (10 ng/mL). **(A)** After 12 h of treatment, cells were harvested, and subsequent measurement of intracellular lactate, succinate, and itaconate was performed through liquid chromatography–high-resolution mass spectrometry. **(B)** Cells were treated and incubated with universally labeled ^13^C glucose ([^13^C_6_]H_12_O_6_) or non-labeled ^12^C glucose for carbon tracing in lactate, succinate, and itaconate through liquid chromatography–high-resolution mass spectrometry. APE was calculated for isotopologues. **(C)** ACOD1 (*Irg1*) expression in activated and treated macrophages was determined by immunoblotting. Original blot was probed for both iNOS and ACOD1/*Irg1* (Figure 1A; Supplementary Figure 1) such that the β-actin control is identical in both figures. *p* < 0.05 when compared to respective control (*), 1400W (‡), LPS (#), LPS + OA (†), and LPS + NO_2_-OA (‡) (Figures 2A,B) using ANOVA; *p* < 0.05 compared to *respective control within M + x* (*), LPS (#), LPS + OA (†), and LPS + NO_2_-OA (‡) using ANOVA (Figure 2C). Data are from n = 2 independent biological experiments, each comprising three technical replicates.

Once the inhibition of NO production was confirmed (Figure 1), intracellular metabolite levels were measured through LC–HRMS at 12 h based on a time course in which RAW 264.7 macrophages were treated with either OA or NO_2_-OA over an 18 h period with and without LPS activation (Supplementary Figure 2). Stimulation of macrophages with LPS increased intracellular lactate, succinate, and itaconate compared to vehicle and control treatments (Figure 2A). Treatment of LPS-activated macrophages with NO_2_-OA reduced the levels of all three metabolites in RAW 264.7 macrophages (Figure 2A) and bone marrow-derived macrophages (BMDMs) at 6 h (Supplementary Figure 3). Pharmacological inhibition of NOS2 with 1400W did not reduce lactate and itaconate concentrations. Moreover, OA had no effect on the levels of these inflammatory metabolites (Figure 2).

Since glycolytic and TCA cycle metabolites were diminished in concentration by NO_2_-OA after LPS activation, we interrogated metabolic shifts using ^13^C tracing and LC–HRMS (Figure 2B). Using universally labeled ^13^C glucose ([^13^C_6_]H_12_O_6_), we examined shifts in energy substrate utilization under the described control and LPS-activated conditions (Supplementary Figure 4A). LPS activation of macrophages resulted in an increased utilization of ^13^C glucose compared to control groups, as indicated by the shift in atomic percent enrichment (APE) from the M+0 to the M+3 isotopologue of lactate (Figure 2B). NO_2_-OA partially inhibited this shift by a ∼5% reduction in the APE of the M+3 isotopologue of lactate, with 1400W not significantly impacting the APE of M+3 lactate (Figure 2B). Interestingly, the APE of the M+0 isotopologue of succinate decreased in the NO_2_-OA treatment group compared to vehicle and 1400W treatment. LPS also decreased M+0 APE compared to control conditions. The M+2 (first turn of the TCA cycle) APE of succinate in NO_2_-OA, LPS + NO_2_-OA, and LPS +1400W treatments was significantly greater than in vehicle or 1400W treatment. NO_2_-OA, both alone and with LPS activation, significantly increased the M+3 APE of succinate compared to vehicle or 1400W treatment or LPS treatment, respectively. The M+4 (second turn of the TCA cycle) APE of succinate was also significantly greater in NO_2_-OA and LPS + NO_2_-OA treatments than in control conditions and LPS alone, indicating that NO_2_-OA preserves the transfer of ^13^C carbons from glutamate and reduces the dilution of ^12^C carbons entering the TCA cycle from other sources (Figure 2B). Although NO_2_-OA reduced the total amount of itaconate after LPS activation, it did not significantly alter the APE of itaconate isotopologues (Figure 2B). Further investigation of itaconate production revealed that NO_2_-OA reduced ACOD1 protein expression in LPS-activated macrophages, whereas 1400W did not (Figure 2C; Supplementary Figure 1). NO_2_-OA and 1400W treatment both inhibited NO production; however, NO_2_-OA has additional mechanisms of action, including the attenuation of pro-inflammatory metabolite concentrations, reduction of the ^13^C carbon dilution of succinate, and inhibition of ACOD1 expression. This reveals that the attenuation of pro-inflammatory mediator expression by NO_2_-OA in LPS-activated macrophages is not solely due to NO_2_-OA inhibition of NOS2 expression and NO production.

### NO_2_-OA inhibits LPS-induced IL-1β expression

**FIGURE 3:**
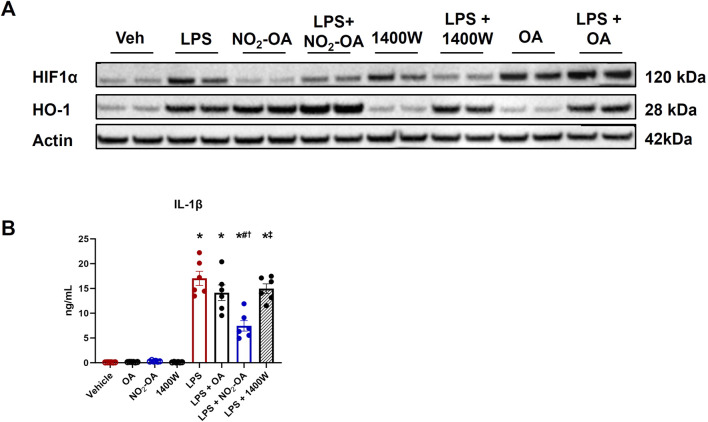
NO_2_-OA treatment, but not 1400W, reduces IL-1β protein expression. Wild-type RAW 264.7 macrophages were treated with vehicle (0.01% DMSO, control), oleic acid (OA, 5 μM, control), NO_2_-OA (5 µM), or 1400W (100 µM), with and without LPS activation (10 ng/mL). **(A)** After 12 h of treatment, HIF1α and HO-1 expressions were measured in cell lysates from treated macrophages through immunoblot (β-actin housekeeping control; Supplementary Figure 1). **(B)** IL-1β from treated cell lysates was measured through ELISA. *p* < 0.05 when compared to respective control (*), LPS (#), LPS + OA (†), and LPS + NO_2_-OA (‡) using ANOVA. Data are from n = 2 independent biological experiments, each comprising three technical replicates.

Lower levels of succinate corresponded with decreased MCP-1 and TNF-α secretion in LPS-activated macrophages treated with NO_2_-OA or 1400W (Figure 1C), indicating that both NO_2_-OA and 1400W may mitigate effector function in inflammatory macrophages by suppressing pro-inflammatory metabolite accumulation and secretion of signaling mediators. Succinate accumulation inhibits prolyl hydroxylases, thus stabilizing HIF1α expression (Selak et al., 2005). This insight motivated the evaluation of HIF1α regulation and expression of the downstream pro-inflammatory cytokine, IL-1β. HIF1α protein expression was increased in LPS-activated macrophages compared to vehicle treatment (Figure 3A; Supplementary Figure 1). Macrophages treated with NO_2_-OA or 1400W upon LPS activation displayed decreased HIF1α protein expression. Surprisingly, macrophages treated only with 1400W resulted in HIF1α stabilization. This result was unexpected as previous studies have shown that NO inhibits prolyl hydroxylases through S-nitrosylation to stabilize HIF1α, and thus, one might hypothesize that treatment with 1400W would result in the degradation of HIF1α as observed after LPS activation (Metzen et al., 2003; Chowdhury et al., 2011; Li et al., 2007). NO_2_-OA, concurrent with LPS activation, further induced an already upregulated heme oxygenase 1 (HO-1) protein expression, whereas 1400W did not engage Keap1/Nrf2-regulated adaptive signaling responses (Figure 3A; Supplementary Figure 1). Although 1400W reduced HIF1α protein expression, ELISA results demonstrated that only NO_2_-OA decreased IL-1β protein expression (Figure 3B). These data reveal that NO_2_-OA induces the limitation of classical, pro-inflammatory polarization via mechanisms partly attributed to the reduction in succinate accumulation and succinate-mediated HIF1α stabilization.

### Deletion of Irg1 reduces the levels of succinate and itaconate, but not IL-1β production

**FIGURE 4:**
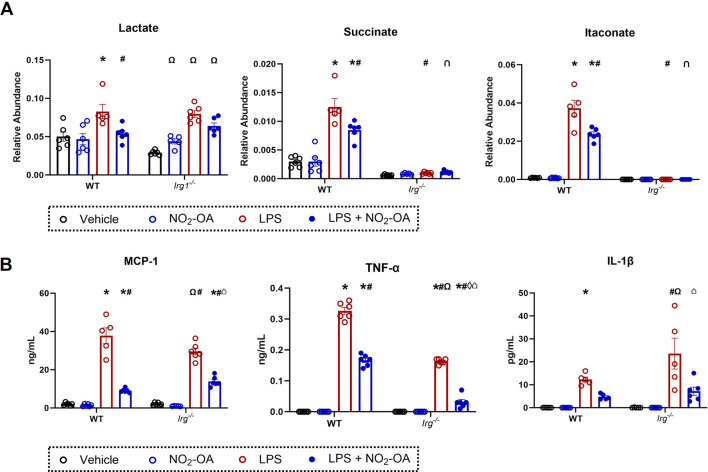
Deletion of *Irg1* reduces levels of succinate and itaconate, but not IL-1β production. Wild-type RAW 264.7 macrophages (WT) and ACOD1^−/−^/*Irg1* ^−/−^ RAW 264.7 macrophages (*Irg1* ^*−/−*^) were treated with vehicle (0.01% DMSO, control), or NO_2_-OA (5 µM) was stimulated with LPS (10 ng/mL) for 12 h prior to harvest. **(A)** Lactate, succinate, and itaconate were measured through liquid chromatography–high-resolution mass spectrometry. **(B)** MCP-1, TNF-α, and IL-1β were measured in treated cells using ELISA. *p* < 0.05 when compared to respective control (*), WT LPS (#), WT LPS + NO_2_-OA (‡), *Irg* ^*−/−*^ vehicle (Ω), *Irg* ^*−/−*^ NO_2_-OA (◊), and *Irg* ^*−/−*^ LPS (⌂) using ANOVA. Data are from n = 2 independent biological experiments, each comprising three technical replicates.

NO_2_-OA lowers itaconate levels in activated macrophages, whereas 1400W did not; however, both NO_2_-OA and 1400W reduced succinate concentrations (Figure 2). These data suggest that the reduction in intracellular succinate by NO_2_-OA is, in part, a result of decreased itaconate levels. More specifically, as ACOD1 protein expression decreases, there is less itaconate available to inhibit SDH activity, thereby reducing succinate accumulation. To test this concept, *Irg1* ^*−/−*^ RAW 264.7 macrophages were used to examine the relationship between itaconate, succinate, and downstream inflammatory signaling (Supplementary Figures 1,5). LPS activation induced a similar increase in lactate levels in WT and *Irg1* ^*−/−*^ macrophages compared to vehicle treatment, although the mean baseline level of lactate in vehicle-treated *Irg1* ^*−/−*^ cells trended lower than that of WT cells (Figure 4A). NO_2_-OA blunted lactate increases to a similar extent in both WT and *Irg1* ^*−/−*^ macrophages. *Irg1* ^*−/−*^ macrophage activation with LPS did not result in an increase in either succinate or itaconate compared to WT macrophages (Figure 4A). A loss of ACOD expression also inhibited LPS-induced cytokine expression, with levels of MCP-1 and TNF-α being lower in *Irg1* ^*−/−*^ macrophages than in WT. NO_2_-OA abrogated MCP-1 and TNF-α expressions after LPS activation in both WT and *Irg1* ^*−/−*^ macrophages and IL-1β expression in LPS-activated *Irg1* ^*−/−*^ macrophages. Intriguingly, IL-1β expression increased in *Irg1* ^*−/−*^ macrophages after LPS activation compared to that in WT (Figure 4B).

### NO_2_-OA reduces macrophage reliance on extracellular glutamine for succinate production

**FIGURE 5:**
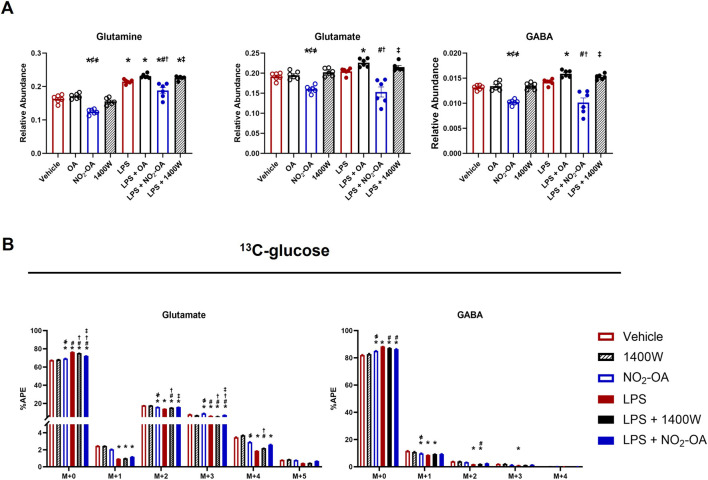
LPS-activated macrophages increased glutamine dependence. Wild-type RAW 264.7 macrophages were treated with vehicle (0.01% DMSO, control), oleic acid (OA, 5 μM, control), NO_2_-OA (5 µM), or 1400W (100 µM) with and without LPS activation (10 ng/mL). **(A)** After 12 h of treatment, cells were harvested, and intracellular glutamine, glutamate, and γ-aminobutyric acid (GABA) were measured using liquid chromatography–high-resolution mass spectrometry. **(B)** To examine shifts in energy substrate utilization under the activation/treatment conditions, cells were incubated with universally labeled ^13^C glucose ([^13^C_6_]H_12_O_6_) normalized to the appropriate ^12^C control. APE was calculated for isotopologues indicating the incorporation of labeling into glutamine, glutamate, and GABA. *p* < 0.05 when compared to respective control (*), OA (¢), 1400W (҂), LPS (#), LPS + OA (†), and LPS + NO_2_-OA (‡) using ANOVA. Data are from n = 2 independent biological experiments, each comprising three technical replicates.

NO_2_-OA treatment of RAW 264.7 macrophages decreased the unlabeled pool of succinate and increased ^13^C-glucose utilization, as evidenced by the increased APE in succinate isotopologues (Figure 2). To better understand how NO_2_-OA directs energy substrate utilization in LPS-activated macrophages, the effects of NO_2_-OA on glutamine reliance in macrophages with and without LPS activation were evaluated. NO_2_-OA induced a significant decrease in the intracellular levels of glutamine, glutamate, and γ-aminobutyric acid (GABA) compared to vehicle-, OA-, and 1400W-treated cells (Figure 5A). Upon LPS activation, NO_2_-OA also reduced levels of these metabolites, whereas 1400W and OA did not (Figure 5A). Then, the APEs of glutamine, glutamate, and GABA were determined following ^13^C-glucose addition (Figure 5B). NO_2_-OA reduced the amount of unlabeled glutamate (M+0) after LPS activation and increased the APEs of M+2, M+3, and M+4 compared to LPS alone (Figure 5B). LPS activation increased the unlabeled pool of glutamate (M+0) and decreased the APEs of the M+2, M+3, and M+4 isotopologues of glutamate. A similar trend was observed for GABA, where LPS activation increased the unlabeled pool of GABA (M+0), with observed reductions in the APEs of M+1, M+2, and M+3 isotopologues (Figure 5B). Collectively, these data indicate an increased reliance on glutamine as a metabolic substrate after LPS activation.

**FIGURE 6:**
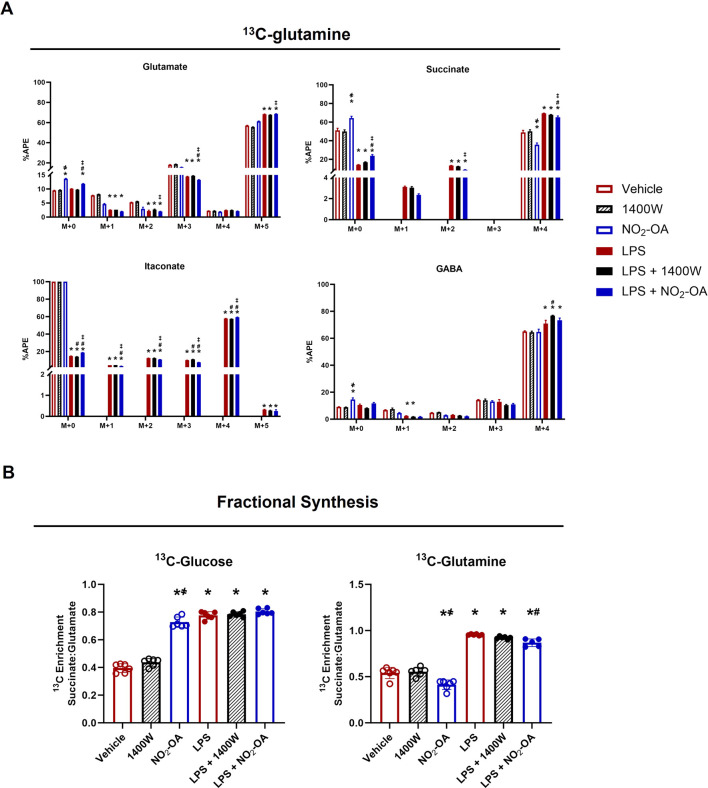
NO_2_-OA reduces glutamine reliance in LPS-activated macrophages. **(A)** RAW 264.7 cells were incubated with ^13^C-glutamine ([^13^C_5_]H_10_N_2_O_3_) and were activated/treated as indicated. APE was calculated for isotopologues of glutamate, succinate, itaconate, and γ-aminobutyric acid (GABA). **(B)** Enrichment of ^13^C in succinate derived from glutamate (fractional synthesis) was calculated for cells utilizing either [^13^C_6_]H_12_O_6_ or [^13^C_5_]H_10_N_2_O_3_ for each treatment group. *p* < 0.05 when compared to respective control (*), 1400W (҂), LPS (#), and LPS +1400W (‡). Data are from n = 2 independent biological experiments, each comprising three technical replicates.

To further investigate the LPS-induced reliance on glutamine, universally labeled ^13^C-glutamine ([^13^C_5_]H_10_N_2_O_3_) was used for the determination of APE in several metabolites (Supplementary Figure 4B). Figure 6 shows the APE for isotopologues of glutamate, succinate, itaconate, and GABA (Figure 6A). As expected, the highest enrichment for glutamate is in the M+5 isotopologue with an increase in M+5 for LPS-activated cells. NO_2_-OA did not significantly reduce enrichment in M+5 glutamate but increased the unlabeled pool (M+0) with concomitant reductions in the APE of M+2 and M+3 isotopologues (Figure 6A). NO_2_-OA also increased the unlabeled pool of succinate (M+0) and significantly reduced the APE of the M+4 isotopologue, while LPS activation demonstrated a reverse effect. NO_2_-OA significantly inhibited this LPS effect by increasing M+0 and decreasing the M+4 pool of succinate after LPS activation, whereas 1400W did not (Figure 6A). Without LPS activation, the intracellular amount of itaconate is very low and is observed only for the M+0 isotopologue. LPS activation significantly increased the production of itaconate, resulting predominantly in the M+4 isotopologue observed from ^13^C-glutamine (Strelko et al., 2011). After LPS activation, NO_2_-OA significantly decreases the APE for M+1, M+2, and M+5 itaconate, but not for M+4. Similar to glutamate, GABA is predominantly enriched in all carbons (M+4) as GABA is the enzymatic product of glutamate decarboxylation (Fenalti et al., 2007). NO_2_-OA increased the unlabeled pool (M+0) of GABA compared to other treatments but did not significantly alter the APE of the M+4 isotopologue, whereas 1400W significantly increased M+4 after LPS activation (Figure 6A).

To compare the effects of NO_2_-OA on metabolism through the TCA cycle, the fractional synthesis of succinate from glutamate was calculated. A ratio of 1 would indicate that the APEs of succinate and glutamate are equal and that no dilution of the ^13^C label has occurred. When ^13^C-glucose was used as the energy substrate, NO_2_-OA increased the ratio of succinate to glutamate enrichment compared to the vehicle and 1400W groups. The enrichment observed by NO_2_-OA treatment approaches the level calculated for LPS activation alone and LPS + NO_2_-OA or LPS +1400W treatment (Figure 6B). When ^13^C-glutamine is used as the energy source, NO_2_-OA reduces the enrichment ratio compared to vehicle and 1400W. LPS increases this ratio to almost 1, indicating minimal dilution of the label and showing that the carbons in succinate are derived from extracellular glutamine via glutamate metabolism. NO_2_-OA treatment after LPS activation significantly reduces this ratio. These data reflect what is observed in the APE of succinate for both ^13^C-glucose and ^13^C-glutamine utilization (Figures 2B, 6A). Collectively, these data demonstrate that NO_2_-OA reduces the absolute levels of pro-inflammatory metabolites and limits reliance on extracellular glutamine for succinate production, both in the presence and absence of LPS activation.

### NO_2_-OA repurposes extracellular glutamine for glutathione synthesis

**FIGURE 7:**
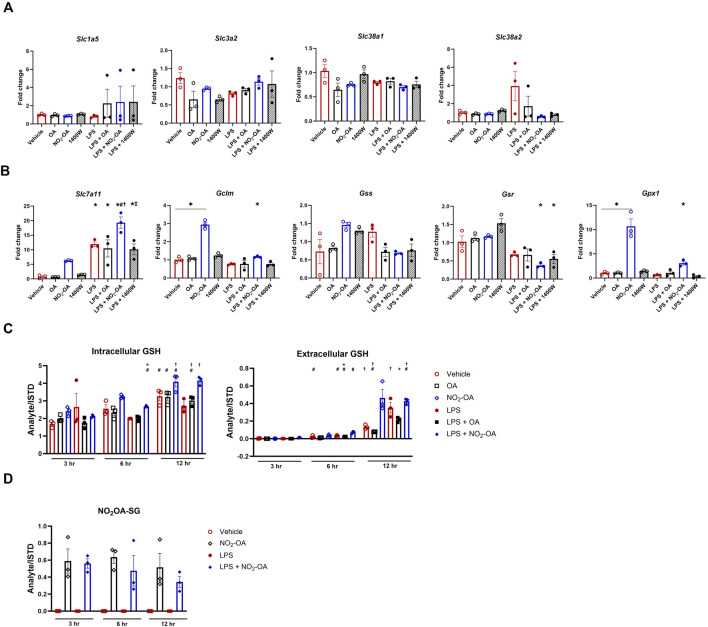
NO_2_-OA repurposes extracellular glutamine for glutathione synthesis. **(A)** Cells were treated with vehicle, OA, NO_2_-OA, and 1400W, with and without LPS activation for 6 h prior to collection in TRIzol™ for RNA preservation. cDNA was prepared, and PCR was performed using primers specific for glutaminase (*GLS*), solute carrier family 1 (neutral amino acid transporter), member 5 (*Slc1a5*), solute carrier family 3, member 2 (*Slc3a2*), solute carrier family 38 member 1 (*Slc38a1*), and solute carrier family 38 member 2 (*Slc38a2).* Data were normalized using GAPDH (control gene) expression and vehicle (control condition) and are reported as fold change (2^-∆∆CT). **(B)** PCR was performed using primers specific for solute carrier family 7 (cationic amino acid transporter, y + system) (*Slc7a11;* i.e., xCT), glutamate–cysteine ligase, modifier subunit (*Gclm*), glutathione synthetase (*Gss*), glutathione reductase (*Gsr*), and glutathione peroxidase 1 (*Gpx1*). Data were normalized using GAPDH (control gene) expression and vehicle (control condition) and are reported as fold change (2^-∆∆CT). **(C)** Treated cells (3–12 h) were collected in N-ethylmaleimide (NEM) derivatization buffer for the quantification of GSH. Media extracted with NEM buffer were also analyzed to determine extracellular GSH by LC–HRMS. **(D)** Media were harvested from treated cells (3–12 h) and subjected to solid-phase extraction to capture GS-OA-NO_2_ for quantification through LC–HRMS (Supplementary Figure 7). Data points are technical replicates of BMDMs pooled from n = 6 mice.

Finally, we sought to understand how NO_2_-OA minimized glutamine utilization in the TCA cycle of RAW 264.7 macrophages with and without LPS activation. First, we examined the gene expression of glutamine transporters, including *Slc38a1*, *Slc38a2*, *Slc1a5*, and *Slc3a2*. There was no difference in gene expression across all treatment groups at 6 h (Figure 7A). Since NO_2_-OA is an electrophile that covalently adducts small thiols and is known to activate the antioxidant response, we investigated GSH synthesis and the alkylation of GSH by NO_2_-OA (Woodcock et al., 2018). Changes were observed in gene expression across various parts of glutathione metabolism. The expression of *Slc7a11* (xCT), the cystine/glutamate antiporter, is upregulated with NO_2_-OA treatment independent of LPS activation. LPS activation also results in an upregulation of *Slc7a11* gene expression with a further upregulation in macrophages activated with LPS and treated with NO_2_-OA (Figure 7B), which was corroborated in BMDMs (Supplementary Figure 6A). NO_2_-OA with and without LPS activation upregulated the expression of *Gclm*, the glutamate–cysteine ligase modifier subunit, along with the expression of *Gpx1*, glutathione peroxidase 1. LPS activation lowered the expression of both glutathione synthetase (*Gss*) and glutathione reductase (*Gsr*), with no rescue from NO_2_-OA treatment (Figure 7B). This contrasted with the upregulation of *Gsr* and *Gss* in BMDMs with NO_2_-OA treatment after LPS stimulation (Supplementary Figure 6A). Intracellular GSH levels are reduced at 6 and 12 h, and extracellular GSH levels are reduced at 12 h with LPS activation in RAW 264.7 macrophages; NO_2_-OA treatment increases both extracellular and intracellular GSH levels at 12 h independent of LPS activation, whereas NO_2_-OA alone increases GSH at 3 h in BMDMs (Figure 7C; Supplementary Figure 6B). NO_2_-OA alkylates GSH through a 1,4- or a 1,6-Michael addition to form GS-OA-NO_2_. GS-OA-NO_2_ was quantified in RAW 264.7 macrophages and BMDM supernatants using stable isotope dilution LC–HRMS (Figure 7D; Supplementary Figure 6C; Supplementary Figure 7).

## Discussion

Nitroalkenes are abundant and reversibly reactive, soft electrophiles that preferentially target kinetically susceptible members of the Cys proteome (Delmastro-Greenwood et al., 2015; Salvatore et al., 2021; Salvatore et al., 2013). This class of mediators is a) basally present in humans and animal models, b) generated endogenously at greater concentrations through metabolic and inflammatory reactions, c) present in tissues and fluids as free, esterified, and nucleophile-adducted species, and d) modeled using pure synthetic homologs that have been developed as potential therapeutic agents. Related to the latter, Phase 2 trials are ongoing in an inflammatory disorder—the treatment of airway hyperreactivity in obesity-related asthma (NCT03762395) (Manni et al., 2021). With macrophages centrally poised as sentinel innate immune cells in many tissue compartments, understanding how small-molecule electrophiles such as NO_2_-OA impact metabolism to control effector function in this cell population has broad implications. This responsiveness to oxidative stress is an evolutionarily conserved mechanism that permits organisms to adapt to changes in metabolic and inflammatory status. The present study defines the connections between fatty acid nitroalkene reactions and the bioenergetic demands of pro-inflammatory macrophages and effector function as it is observed that targeting immune cell metabolism represents a therapeutic intervention point for a myriad of inflammatory diseases. In this study, we identify NO_2_-OA as a mediator of pro-inflammatory metabolic responses in LPS-activated murine macrophages, acting via the suppression of intracellular succinate accumulation as a result of the downregulation of ACOD1 protein expression and (Palsson-McDermott et al., 2015) the redirection of extracellular glutamine carbons toward glutathione synthesis. This metabolic phenotype, induced by small-molecule nitroalkenes, may account for the broad spectrum of reported anti-inflammatory and pro-resolving actions of these and related electrophilic mediators, both *in vitro* and *in vivo*.

The mechanisms accounting for NO_2_-OA-mediated reduction of ACOD1 protein expression in activated macrophages remain to be fully elucidated. The production of ACOD1 is associated with LPS-mediated activation of protein kinase C (PKC) signaling in macrophages (Hu et al., 2022). Furthermore, inhibition of branched-chain amino acid transferase 1 (BCAT1) with the leucine analog ERG240 downregulates ACOD1 mRNA and decreases protein levels and intracellular itaconate levels in LPS-activated macrophages (Zhang et al., 2022). Relevant to the mechanism of action of NO_2_-OA, both PKC and BCAT1 contain redox-reactive Cys-rich motifs (Steinberg, 2015), and nitroalkene derivatives of oleic, linoleic, and arachidonic acids are reported to inhibit inflammatory cell PKC activity (Bonilla et al., 2013; Bates et al., 2011). BCAT1 is also susceptible to oxidative modifications that inhibit enzymatic activity (Coles et al., 2009). Thus, the lower levels of ACOD1 observed herein may be due to NO_2_-OA alkylation of Cys, leading to altered function or inactivation of BCAT1. Chemi-proteomic identification of the global Cys proteome susceptible to alkylation by NO_2_-OA in basal and activated macrophages is planned and can better define this aspect of small-molecule nitroalkene inflammation resolution.

Increases in HIF1α expression or its stabilization also increases ACOD1/*Irg1* expression. It has been reported that increases in itaconate result in the activation of the Nrf2-regulated antioxidant response (Mills et al., 2018). It is noted that this *ex vivo* effect required a membrane-permeable itaconate derivative, 4-octyl-itaconate, being applied at high concentrations in cell culture (Mills et al., 2018; Ryan and O'Neill, 2020). Under the control of Nrf2, HO-1 is upregulated in LPS-activated macrophages, a known defense mechanism to physiologically adapt to oxidant challenge (Naito et al., 2014). HO-1 protein expression increases in response to NO_2_-OA treatment under conditions where itaconate levels are diminished. As NO_2_-OA is a kinetically more reactive electrophile than α, β-unsaturated ketones such as itaconate, it may outcompete itaconate to alkylate Keap1 and release Nrf2 to translocate to the nucleus. Similarly, *Gpx1* is transcriptionally controlled in response to oxidative stress; however, in contrast to HO-1, *Gpx1* regulation by Nrf2 has not been confirmed, indicating a potential novel NO_2_-OA-mediated post-translational modification regulating glutathione metabolism (Lubos et al., 2011).

Itaconate inhibits SDH, which leads to succinate accumulation, a metabolic hallmark of inflammation (Lampropoulou et al., 2016). As mitochondrial succinate accumulates in excess in the cytosol via the dicarboxylic acid transporter (SLC25A10) in the inner mitochondrial membrane and the voltage-dependent anion channel in the outer mitochondrial membrane, prolyl hydroxylase enzyme activity becomes impaired, and HIF1α activity is stabilized in a metabolic state of “pseudo-hypoxia.” Herein, HIF1α protein levels are lower in LPS-activated macrophages treated with NO_2_-OA and 1400W than in macrophages treated with LPS alone. This occurs concurrently with the reduction in intracellular succinate being induced by NO_2_-OA and 1400W treatment. The resulting destabilization of HIF1α protein levels may thus contribute to the NO_2_-OA-induced reduction in intracellular lactate and pro-inflammatory cytokines. In aggregate, these data support the concept that small-molecule nitroalkenes can attenuate the highly glycolytic and inflammatory phenotype mediated by HIF1α under conditions of pseudo-hypoxia.

The lack of ACOD protein expression in the *Irg1* ^*−/−*^ macrophage prevents itaconate production in the presence of LPS, preserving energetic flux through the TCA cycle (Figure 4A). However, LPS-stimulated WT and *Irg1* ^*−/−*^ cells have decreased intracellular lactate upon NO_2_-OA treatment, supporting an anti-inflammatory action independent of ACOD1 and itaconate, thus ultimately decreasing the energetic burden in response to stimuli. Additionally, the lack of ACOD1/*Irg1* resulted in less MCP-1 and TNF-α secretions in LPS-activated *Irg1* ^*−/−*^ cells than in WT, while NO_2_-OA treatment in both activated WT and *Irg1* ^*−/−*^ cells reduced the levels of MCP-1 to a similar extent. Downregulated pro-inflammatory cytokine production in activated *Irg1* ^*−/−*^ macrophages following treatment with NO_2_-OA also indicates an anti-inflammatory mechanism independent of ACOD1 and itaconate.

In LPS-activated macrophages, succinate accumulation occurs concurrently with SDH inactivation, indicating that succinate can accumulate through mechanisms other than SDH inhibition (Lee et al., 1995). Herein, NO_2_-OA reduced ACOD1 expression, but 1400W did not, yet both agents reduced succinate levels after LPS activation (Figure 2). Glutamine metabolism is an alternative route to succinate accumulation in LPS-stimulated macrophages. In the present study, NO_2_-OA reduced succinate accumulation and attenuated inflammatory indices by reducing cellular metabolic reliance on extracellular glutamine—regardless of inflammatory status. Treatment with 1400W with or without LPS activation did not reduce intracellular levels of glutamine or glutamate (Figure 5). The transporters *Slc1a5*, *Slc38a1*, and *Slc38a2* allow for external cytosolic glutamine transport followed by SLC1A5 transport into the mitochondria (Scalise et al., 2017). Glutamine is a vital alternative fuel source and substrate for the synthesis of nucleotides, non-essential amino acids, and GSH, and it is rapidly depleted under cellular stress and hypoxic conditions (Yoo et al., 2020). This hallmark metabolic shift is observed in cancer cells and warrants further investigation in the context of inflammatory-activated macrophages. Analysis of glutamine transporter gene expression revealed that no significant changes occurred across any of the treatment groups. Rather, NO_2_-OA, both with and without LPS activation, redirects glutamine utilization toward GSH synthesis. Although NO_2_-OA upregulates the antioxidant response, as evidenced by the upregulation of *Slc7a11* and *Gclm*, it concurrently alkylates GSH to produce NO_2_-OA-SG adducts, thus utilizing the substrate it helps generate. NO_2_-OA also upregulates *Gpx1* gene expression (Figure 7). GPX1 reduces hydroperoxides to water and is part of the antioxidant defense (Lubos et al., 2011). The upregulation of GSH synthesis and expression of *Gpx1* do not occur with 1400W treatment; therefore, other pathways to reduce succinate accumulation and inflammation using NO_2_-OA are possible. More robust upregulation of the antioxidant defense system regulators *Gsr* and *Gss* was found in BMDMs, corroborating the mitigation of inflammation by NO_2_-OA in both lineages of macrophages (Supplementary Figure 6).

The post-translational modification of proteins expands the functional proteome over 50-fold beyond those that are genetically encoded, with 10% of all proteins undergoing diverse forms of enzyme-catalyzed and non-catalytic lipidation reactions. The alkylation of nucleophilic amino acids, predominantly Cys, using endogenous and synthetic small-molecule nitroalkenes not only modifies protein–Cys structural and functional properties but also impacts protein localization, trafficking, stability, and signaling actions. The present studies reveal that NO_2_-OA alkylates key metabolic proteins and redirects metabolism to reduce pro-inflammatory macrophage effector functions. This provides a foundation for understanding whether endogenous electrophilic fatty acid nitroalkenes are sufficient in concentration to alter antigen presentation and cross-presentation such that changes in innate immune cell function translate to changes in adaptive immunity and promotion of inflammation resolution. At the same time, this can reveal whether changes in endogenously generated or orally administered small-molecule nitroalkene levels are sufficient to attenuate the highly glycolytic and disrupted TCA cycle of activated macrophages in an animal model of inflammation. Overall, the present report expands and reinforces the pleiotropic signaling actions of electrophilic nitroalkenes by demonstrating that targeting immune cell metabolism to attenuate pro-inflammatory metabolic intermediate concentrations can be a viable intervention for treating chronic inflammatory disorders.

### Experimental procedures

#### Immortalized cell culture and reagents

Wild-type and *Irg1* ^−/−^ RAW 264.7 (ATCC, Manassas, VA, United States) murine macrophages were grown in Gibco Dulbecco’s modified Eagle medium (DMEM, A1443001) supplemented with 10% heat-inactivated fetal bovine serum (FBS, Gibco, Grand Island, NY, A5669801) for all studies unless otherwise specified. To generate *Irg1* ^*−/−*^/*ACOD1* ^−/−^ cells, wild-type RAW 264.7 cells (ATCC) were transfected with mouse IRG1 CRISPR/Cas9 KO plasmid and mouse IRG1 HDR plasmid (Santa Cruz, #sc-421149, Dallas, TX, United States) using the transfection reagent Viromer RED (Lipocalyx GmbH, Germany) at a 1:1 ratio with selection for clones using puromycin. Clones were confirmed using real-time quantitative PCR and Western blotting for expression. RAW 264.7 *Irg1* ^*−/−*^/*ACOD1* ^−/−^ cells were grown in DMEM (10% FBS, 10 μg/mL puromycin (Gibco, A1113803). *Klebsiella pneumoniae* was purchased from ATCC (strain 43816, serotype 2). LPS was obtained from Sigma, St. Louis, MO, United States (L4391, Lot 067M4036V). NO_2_-OA was synthesized as previously described (Woodcock et al., 2013), and OA was purchased from Cayman Chemical Company, Ann Arbor, Michigan, United States (90410). The NOS2 pharmacological inhibitor, 1400W dihydrochloride, was purchased from Sigma (W4262).

#### Immortalized cell treatments

RAW 264.7 cells plated at 1 × 10^6^ cells/well in six-well plates were cultured for 12 h (37 °C, 5% CO_2_) in 10% FBS/DMEM and treated with or without LPS (10 ng/mL), NO_2_-OA (5 µM), 1400W (100 µM), and OA (5 µM) in 5% FBS/DMEM. The cells were harvested at 0, 6, 12, or 18 h post-treatment for the analysis of intracellular metabolites and cytokine levels in the media. For labeling studies, cells were cultured for 12 h in DMEM containing universally labeled ^13^C glucose ([^13^C_6_]H_12_O_6_) and ^13^C-glutamine ([^13^C_5_]H_10_N_2_O_3_) (CLM-481-PK and CLM-1166-PK; Cambridge Isotopes Laboratories) prior to metabolite extraction (Supplementary Figure 4). For *Irg1* ^*−/−*^/*ACOD1* ^−/−^ studies, the *K. pneumoniae* (”+“) supernatant was prepared as previously described (van der Geest et al., 2023). Cells were incubated for 6 h with 5% (v/v) *K. pneumoniae* supernatants as a positive control to confirm ACOD1 expression (Supplementary Figure 5).

#### Primary cell collection, culture, and treatment

Male 5–7-week-old C57BL/6J mice (Jackson Laboratories, Bar Harbor, ME) were housed under standard conditions (12:12 light: dark cycle, 65°F–75°F, food/water *ad libitum*). Mice were humanely euthanized (University of Pittsburgh IACUC-approved protocol #23012457) via lethal injection of sodium pentobarbital solution (Fatal-Plus, Vortech Pharmaceuticals, Dearborn, MI). Femurs and tibias were harvested as previously described (Toda et al., 2021). In brief, isolated bones were severed and perfused with at least 5 mL of cold 2% HI-FBS in PBS through a 20-gauge needle to collect bone marrow-derived cells. After pooling, cells were pelleted, and erythrocytes were lysed with ACK lysis buffer (Gibco, Grand Island, NY). After sequential passage through 70 μM and 40 μM sterile strainers, cells were pelleted by centrifugation, and cell viability was assessed using Trypan Blue solution (0.4%, Gibco, Grand Island, NY) exclusion on a hemocytometer. Cells were seeded at a density of 1 × 10^6^ live cells/well on untreated six-well multi-dishes in 10% FBS/1% penicillin–streptomycin DMEM media supplemented with 2 mM glutathione (Thermo Fisher Scientific, Waltham, MA) and 20 ng/mL macrophage colony-stimulating factor (M-CSF; Stem Cell Technologies, Vancouver, Canada) to promote monocyte to BMDM differentiation. Media were exchanged every other day and on day 7, the FBS concentration was reduced to 5% for the treatment of BMDMs. BMDMs were treated with compounds and the concentrations outlined in “*Immortalized cell treatments*” above. BMDMs were collected at 3, 6, and 18 h post-treatment for measurement of intracellular polar metabolites and RNA, and media were collected to measure GS-OA-NO_2_ adduct formation.

#### Metabolite measurement

For liquid chromatography–high resolution mass spectrometry analysis, RAW 264.7 cells were plated in DMEM (10% FBS) for 12 h, and then +/-LPS (10 ng/mL), NO_2_-OA (5 µM), 1400W (100 µM), and OA (5 µM) were added for various times in 5% FBS/DMEM. Following treatment, the supernatant was removed, and the cells were washed with cold PBS. PBS was aspirated, and cells were rapidly lysed with cold 80% methanol (*aq*) containing 100 µM of each standard (lactate-d_3_, alanine-d_3_, creatinine-d_3_, and taurine-d_4_). Cell lysates were removed from the plate using cell scrapers. Samples were vortexed and centrifuged for 10 min at 21,000 x g for 10 min at 4 °C. The supernatant was removed from the precipitated protein and cell debris. Samples were injected via a Thermo Vanquish UHPLC and separated using a reversed-phase Thermo HyperCarb Porous Graphite Column (2.1 × 100 mm, 3 μm particle size) maintained at 55 °C. For the 20-min LC gradient, the mobile phase consisted of the following: solvent A (water/0.1% FA) and solvent B (ACN/0.1% FA). The gradient was as follows: 0–1 min 1% B, increasing to 15% B over 5 min, followed by an increase to 98% B over 5 min. The gradient is held at 98% B for 5 min, followed by equilibration under initial conditions (1% B) for 5 min. The Thermo ID-X Tribrid Mass Spectrometer was operated in both positive and ion modes, scanning in ddMS^2^ mode (2 μscans) from 70 to 800 m*/z* at 120,000 resolution, with an AGC target of 2e^5^ for full scan, 2e^4^ for MS^2^ scans using HCD fragmentation at stepped collision energies of 15, 35, and 50. The source ionization setting was 3.0 and 2.4 kV spray voltage for positive and negative modes, respectively. Source gas parameters were set to 35 for sheath gas, 12 for auxiliary gas at 320 °C, and 8 for sweep gas. Calibration was performed prior to analysis using the Pierce™ FlexMix™ Ion Calibration Solutions (Thermo Fisher Scientific, Waltham, MA). Integrated peak areas were then extracted manually using Quan Browser (Thermo Fisher Xcalibur ver. 2.7) and compared to in-house standard libraries for targeted analysis. Data are reported as relative abundance (peak area of analyte divided by the peak area of the stable isotope-labeled internal standard).

#### ^13^C tracing

For ^13^C experiments, RAW 264.7 cells were plated in DMEM-depleted of either glucose or glutamine and supplemented with either universally labeled ^13^C glucose ([^13^C_6_]H_12_O_6_) or universally labeled ^13^C-glutamine ([^13^C_5_]H_10_N_2_O_3_) to levels standard in glucose- and L-glutamine-supplemented media (4,500 mg/L) (Supplementary Figure 3). Each condition was normalized to its own ^12^C-glucose or ^12^C-glutamine-supplemented control media for a set of corresponding ^12^C treatments that were used to calculate APE for metabolite isotopologues (M + x) measured using LC–HRMS. Data were analyzed using the MIMOSA method (Alves et al., 2015). LC–HRMS conditions described above were utilized for all ^13^C tracing experiments.

#### Glutathione and NO_2_-OA–glutathione (GS-OA-NO_2_) conjugate measurements

RAW 264.7 cells were seeded and treated as previously described above for 3, 6, or 12 h. Culture media was removed and frozen (−80 °C) for downstream analysis. For GSH analysis, cells were washed twice with PBS before the addition of 25 mM N-ethylmaleimide (NEM) in PBS for 15 min (37 °C). NEM derivatization buffer [100 mM NEM (Sigma, 3,876), 50 mM NaCl, 40 mM HEPES (Sigma, H3375), 1 mM EDTA (Sigma, E9884), 20 µM [^13^C_2_^15^N]-GSH (Cambridge Isotope Laboratories)] was added to cells and incubated at room temperature for 30 min. The supernatant was collected through centrifugation at 16,000 x g for 5 min (4 °C). Cell culture media was incubated 1:1 with NEM derivatization buffer and centrifuged as described above. GSH was measured using a Vanquish UHPLC coupled to an Exploris 240 hybrid mass spectrometer (Thermo Fisher Scientific). GSH was retained on a Phenomenex Luna C18 (2.1 × 100 mm) using H_2_O with 0.1% formic acid (Solvent A) and acetonitrile with 0.1% formic acid (Solvent B). The gradient was run at a flow rate of 0.3 mL/min, starting at 5% B and increasing to 15% B at 5 min. The organic phase was then increased to 98% B at 10 min and held for 5 min, followed by equilibration at 5 min under initial conditions. The Thermo 240 hybrid mass spectrometer was operated in positive ion mode, scanning in ddMS^2^ mode (2 µscans) from 70 to 800 m*/z* at 120,000 resolution, with an AGC target of 2e^5^ for full scan and 2e^4^ for MS^2^ scans using HCD fragmentation at stepped collision energies of 15, 35, and 50. The source ionization setting was 3.5 kV spray voltage. Source gas parameters were set to 45 for sheath gas, 12 for auxiliary gas at 320 °C, and 3 for sweep gas. The GSH–NEM peak area was normalized to the stable isotope-labeled internal standard with matching retention time. GS-OA-NO_2_ adducts were isolated via solid-phase extraction on 1 mL C18 columns (Supelco Discovery® DSC-18 SPE tubes).

A stable isotope-labeled GS-OA-NO_2_ internal standard was synthesized by reacting 100 mM [^13^C_2_^15^N] GSH and 100 µM NO_2_-OA in 50 mM phosphate buffer for 3 h at 37 °C. The reaction product was dried under N_2_ and reconstituted in methanol for C18 solid-phase extraction as previously described (Woodcock et al., 2018). [^13^C_2_^15^N]GS-OA-NO_2_ generation was validated through LC–HRMS and added as an internal standard to quantify GS-OA-NO_2_ in the RAW 264.7 supernatant (Supplementary Figure 4). All samples were prepared for LC–HRMS analysis by dilution in methanol and analyzed using a Vanquish UHPLC coupled to an Exploris 240 Hybrid Mass Spectrometer. The GS-OA-NO_2_ adduct was separated on a Phenomenex Luna C18 using H_2_O and 0.1% acetic acid (Solvent A) and acetonitrile and 0.1% acetic acid (Solvent B) at a flow rate of 0.3 mL/min. Solvent B was held at 20% for the first 5 min and increased to 98% over the next 20 min. The wash was held for 4.5 min, followed by equilibration at 20% for 5 min. Exploris 240 source parameters were the same as described above for GSH, except that sheath gas was set to 50, auxiliary gas to 10, and sweep gas to 1. The ddMS^2^ used HCD fragmentation and stepped collision energies of 20, 35, and 50. The GS-OA-NO_2_ peak area was normalized to the stable isotope-labeled internal standard.

#### Cytokine measurements

The cell culture media were collected following treatment with LPS, NO_2_-OA, 1400W, or OA, as described above. Cytokines were quantified using specified enzyme-linked immunosorbent assay (ELISA) kits: MCP-1 (88–7,391–88), TNF-α (88–7,324–88), and IL-1β (88–7,013–77) kits were obtained from Invitrogen, Carlsbad, CA, United States. Samples were diluted so that responses fell within the linear range of the standard curve.

#### Nitrite measurement

Culture media were harvested from treated cells and assessed for nitrite using Griess reagent (G7921, Invitrogen), according to the manufacturer’s instructions. Absorbance (λ = 548) was measured spectrophotometrically using a microplate reader. Nitrite (µM) was quantified within the linear range of the calibration curve generated with sodium nitrite standards.

#### Gene expression

Media were aspirated, and treated cells were washed twice with cold PBS. TRIzol™ reagent (Invitrogen, 15596026) was added to preserve RNA. Total RNA was isolated using a modified phenol–chloroform extraction according to the manufacturer’s guidelines. cDNA was synthesized from template RNA (1 µg) using the iScript cDNA Synthesis Kit (Bio-Rad Laboratories, Ann Arbor, MI, United States, 1708891). mRNA expression was measured using RT-qPCR with TaqMan Fast Advanced Master Mix (Applied Biosystems, Foster City, CA, United States). FAM-dye-labeled TaqMan Gene Expression Assays (Applied Biosystems) were used for target genes of interest in tandem with VIC-dye-labeled *GAPDH* (Applied Biosystems, 4352339E) as the control gene. The following gene expression assays were performed on isolated mRNA from treated cells: *Gclm* (Mm01324400_m1), *Gls* (Mm01257297_m1), *Gpx1* (Mm04207457_g1), *Gss* (Mm00515065_m1), *Gsr* (Mm00439154_m1), *Slc1a5* (Mm00436603_m1), *Slc3a2* (Mm00500521_m1), *Slc7a11* (Mm00442530_m1), *Slc38a1* (Mm00506391_m1), and *Slc38a2* (Mm00628416_m1). Reactions were prepared according to the manufacturer’s specifications and amplified for 40 cycles using the CFX Opus 384 Real-Time PCR System (Bio-Rad Laboratories). Data were collected using CFX Maestro software (v 2.3, Bio-Rad Laboratories) and were normalized using *GAPDH* expression in each sample and “vehicle” as the control condition using the 2^-∆∆CT (fold change) method (Livak and Schmittgen, 2001).

#### Immunoblotting

Cell lysates were prepared by scraping cells into ice-cold radioimmunoprecipitation assay buffer (150 mM NaCl, 1% NP-40, 0.5% sodium deoxycholate, 0.1% SDS, 50 mM Tris, pH 8) with broad-spectrum protease (Pierce, Dallas, TX, United States) and phosphatase inhibitors (Roche, Basal, Switzerland), and the protein concentration was determined using the Pierce BCA Protein Assay Kit (Thermo Fisher). Protein samples were incubated with NuPAGE Sample Buffer and reducing agents at 95 °C for 10 min and loaded onto a NuPAGE Tris-glycine gel. Following electrophoresis, the protein was transferred to a PVDF membrane and incubated with specified antibodies to detect total protein levels: NOS2 (Cell Signaling: 13120); ACOD1 (PA5-102893, Invitrogen; ab222411, Abcam, Waltham, MA, United States); HIF1α (127309; GeneTex, Irvine, CA, United States); HO-1 (26416; Cell Signaling Technology, Danvers, MA, United States); IL-1β (12,242; Cell Signaling Technology); and β-actin (MA5-15739; Invitrogen). Full blot images and densitometry are provided in the Supplemental Material (Supplementary Figure 1).

#### Statistics

The mean ± standard error of the mean (SEM) is shown in all experiments. Normality tests (Shapiro–Wilk) were performed prior to statistical analysis, and non-parametric tests were utilized when abnormal distributions were observed. Student’s t-tests were utilized for comparisons between two groups, while one-way or two-way ANOVA was used for multiple comparisons (Tukey’s test for two-way or normally distributed one-way and Sidak’s test for non-parametric one-way) of two or more variable-containing experiments across groups. GraphPad Prism (Version 10.0.3) was used for all tests. Data are from two independent biological experiments, each comprising three technical replicates unless otherwise noted.

## Data Availability

The original contributions presented in the study are included in the article/Supplementary Material; further inquiries can be directed to the corresponding author.

## Supplementary material

SUPPLEMENTARY FIGURE S1 Full immunoblot images presented in the manuscript. Full immunoblot images showing target protein and housekeeping protein (β-actin) and corresponding densitometry for cropped images in Figures 1A, 2C, 3A; Supplementary Figure 5. ImageJ (NIH) was used to quantify band density. Band density was calculated as target band density/control (β-actin) band density.

SUPPLEMENTARY FIGURE S2 Significant differences in metabolite accumulation occur 12–18 h post-activation/treatment. Intracellular **(A)** lactate, **(B)** succinate, and **(C)** itaconate were measured using liquid chromatography–high-resolution mass spectrometry at 0, 6, 12, and 18 h post-treatment. *p*< 0.05 when compared to respective control (*), LPS (#), and LPS + OA (†).

SUPPLEMENTARY FIGURE S3 NO_2_-OA reduces pro-inflammatory metabolite production in LPS-activated BMDM. **(A)** BMDMs were treated with vehicle (0.01% DMSO, control) or NO_2_-OA (5 µM) with and without LPS activation (10 ng/mL) for 6 h prior to harvest. Itaconate, succinate, glutamine, and glutamate were measured using liquid chromatography–high-resolution mass spectrometry. Data points are technical replicates of BMDMs pooled from n = 6 mice.

SUPPLEMENTARY FIGURE S4 Confirmation of ^13^C enrichment of glucose and glutamine substrates in RAW 264.7 macrophages. Atomic percent enrichment (APE) was calculated for **(A)** ^13^C glucose ([^13^C_6_]H_12_O_6_) and **(B)** ^13^C-glutamine ([^13^C_5_]H_10_N_2_O_3_) under treatment conditions. *p*< 0.05 when compared to control (*).

SUPPLEMENTARY FIGURE S5 Confirmation of *Irg1−/−* in RAW 264.7 macrophages. WT RAW 264.7 macrophages and *Irg1−/−* were activated with *Klebsiella* to demonstrate the knockdown efficiency of ACOD1 expression. The *Klebsiella pneumoniae* (“+”) supernatant was prepared as previously described (van der Geest et al., 2023). Cells were incubated for 6 h with the 5% (v/v) *Klebsiella pneumoniae* supernatant as a positive control to confirm ACOD1 expression.

SUPPLEMENTARY FIGURE S6 NO_2_-OA repurposes extracellular glutamine for glutathione synthesis in BMDMs. **(A)** BMDMs were treated with vehicle (0.01% DMSO, control) or NO_2_-OA (5 µM) with and without LPS activation (10 ng/mL) for 6 h prior to collection in TRIzol™ for RNA preservation. cDNA was prepared, and PCR was performed using primers specific to the modifier subunit (*Gclm*), glutathione peroxidase 1 (*Gpx1*), glutathione reductase (*Gsr*), glutamate–cysteine ligase, glutathione synthetase (*Gss*), and solute carrier family 7 (cationic amino acid transporter, y + system) (*Slc7a11;* i.e., xCT). Data were normalized using GAPDH (control gene) expression and vehicle (control condition) and are reported as fold change (2^-∆∆CT). **(B)** Treated cells (3 h) were collected in N-ethylmaleimide (NEM) derivatization buffer for the quantification of GSH. **(C)** Media were harvested from treated cells (3 and 18 h) and subjected to solid-phase extraction to capture GS-OA-NO_2_ for quantification through LC–HRMS. Data points are technical replicates of BMDMs pooled from n = 6 mice.

SUPPLEMENTARY FIGURE S7 Confirmation of GS-OA-NO_2_ formation in RAW 264.7 macrophages through LC–HRMS. **(A)** The top panel shows the extracted ion chromatogram for GS-OA-NO_2_ that formed after treatment with 5 µM NO_2_-OA. The stable isotope-labeled internal standard [^13^C_2_^15^N]-GS-OA-NO_2_ used to confirm the peak retention time. The lower panel shows the product ion spectra for GS-OA-NO_2_ (*m/z* 635.3315) that was measured from the RAW 264.7 macrophage supernatant. **(B)** Cells treated with LPS do not form the GS-OA-NO_2_ conjugate (top panel). The bottom panel shows the product ion spectra for the stable isotope-labeled internal standard (*m/z* 638.3356), which is 3 amu larger ([^13^C_2_^15^N]-GS-OA-NO_2_) than the endogenously formed GS-OA-NO_2_. Corresponding diagnostic ion pairs include *m/z* 638.3356/635.3315, *m/z* 509.2930/506.2891 (neutral loss of 129 amu from GSH), *m/z* 462.2921/459.2886, and *m/z* 165.0256/162.0219.
