# Supplementary material for: Nitroalkene inhibition of pro-inflammatory macrophage effector function via modulation of signaling metabolite levels
Source: Front Physiol. 2025 Oct 16;16:1426102. doi: 10.3389/fphys.2025.1426102 (PMC12572803; doi:10.3389/fphys.2025.1426102)
Supplement: Supplementary file 1 [file DataSheet1.docx]

**
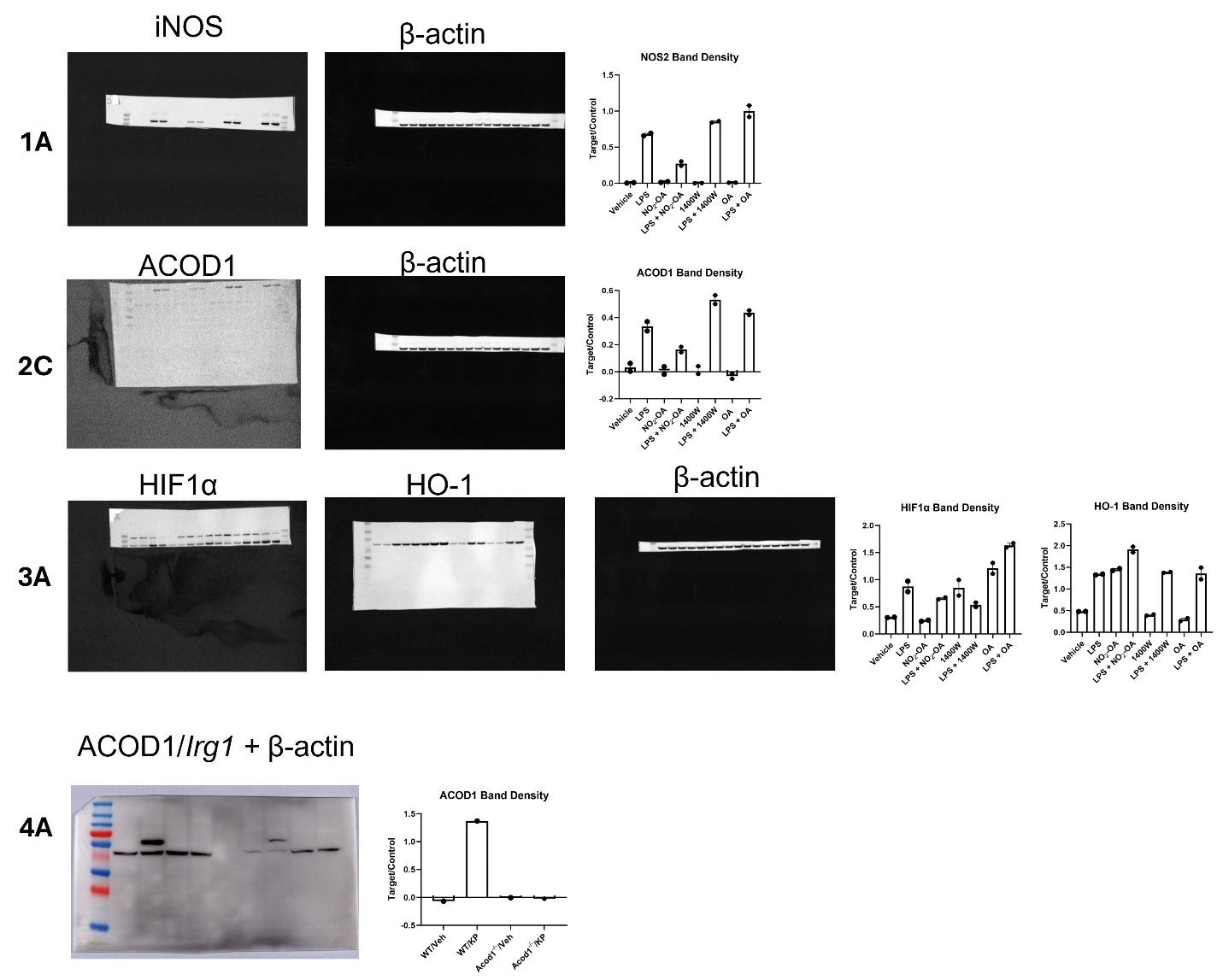
Supplemental Figure 1. Full immunoblot images presented within manuscript.** Full immunoblot images showing target protein and housekeeping protein (β-actin) and corresponding densitometry for cropped images in Figures 1A, 2C, 3A, and Supplemental Figure 5. Image J (NIH) was used to quantify band density. Band density was calculated as target band density/control (β-actin) band density.

**
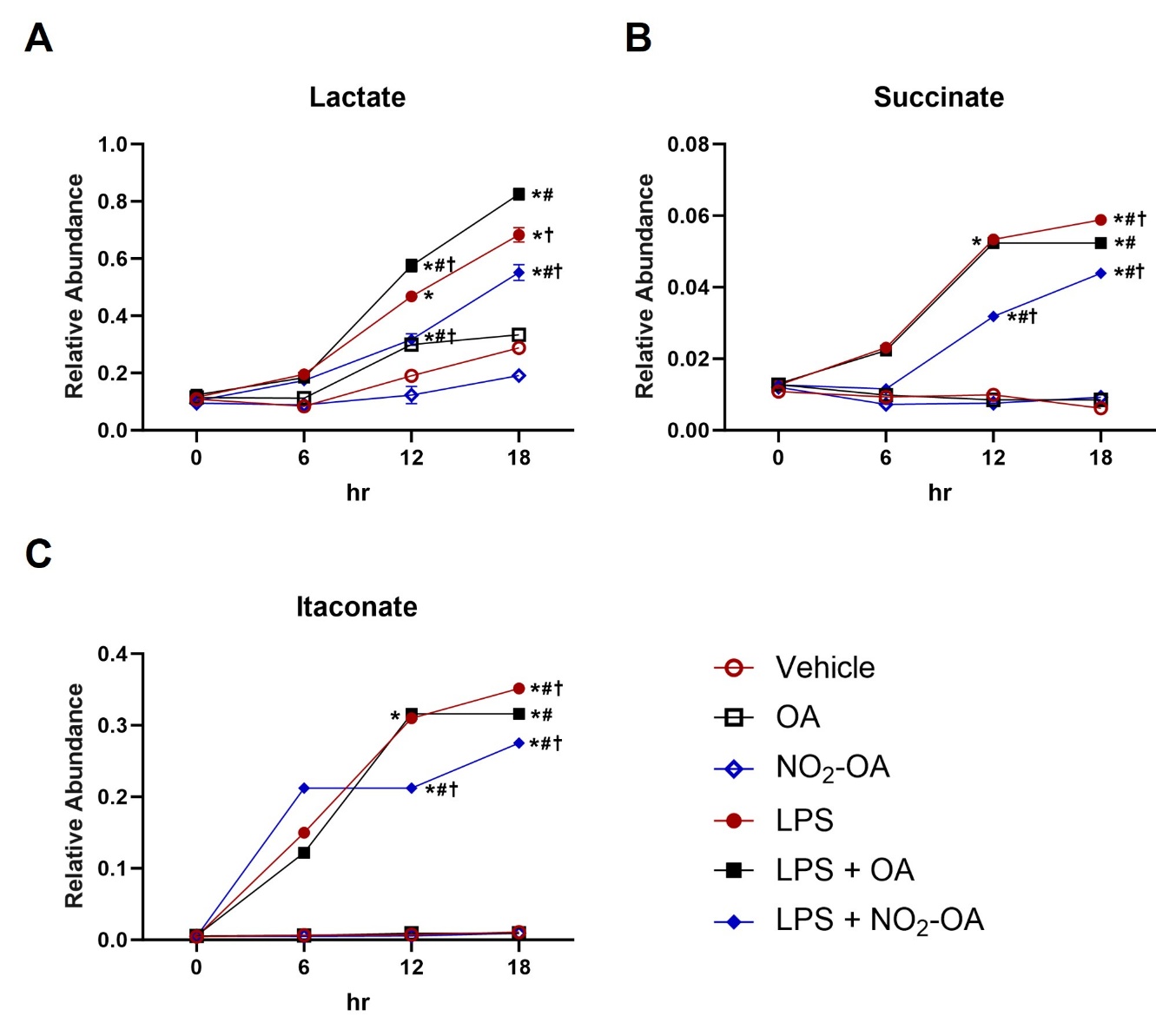
Supplemental Figure 2.** **Significant differences in metabolite accumulation occur 12-18 hr post-activation/treatment.** Intracellular (**A**) lactate, (**B**) succinate, and (**C**) itaconate were measured by liquid chromatography high resolution mass spectrometry at 0, 6, 12, and 18 hr post-treatment. p<0.05 when compared to respective control (*), LPS (#), LPS+ OA (†).

**
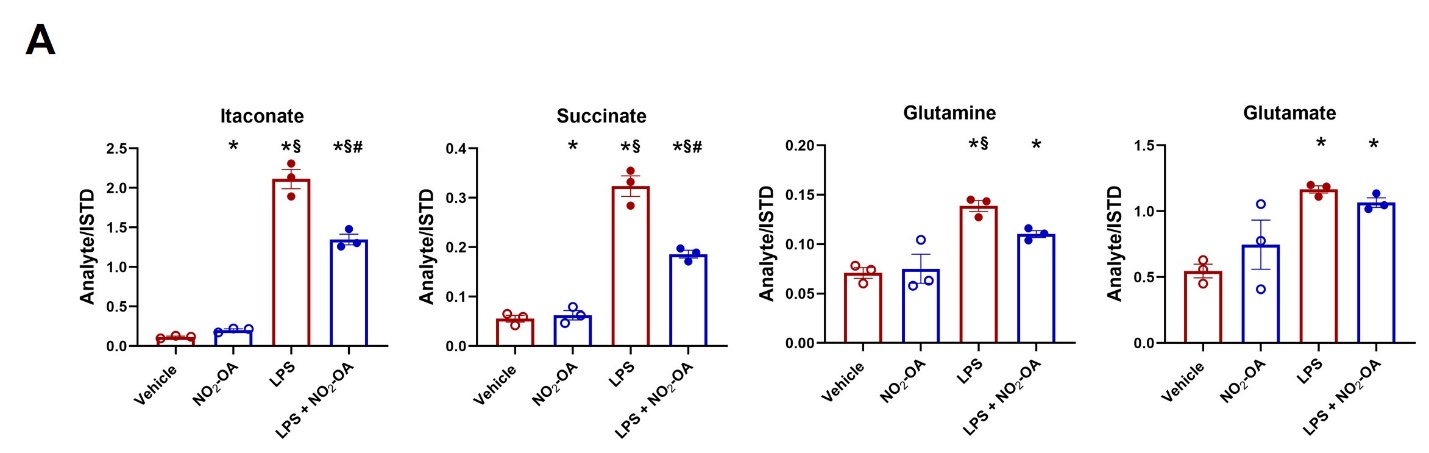
Supplemental Figure 3. NO_2_-OA reduces pro-inflammatory metabolite production in LPS-activated BMDM. (A)** BMDM were treated with vehicle (0.01% DMSO, control), or NO_2_-OA (5 µM) with and without LPS activation (10 ng/mL) for 6 hr prior to harvest. Itaconate, succinate, glutamine, and glutamate were measured by liquid chromatography high-resolution mass spectrometry. Data points are technical replicates of BMDM pooled from n = 6 mice.

**
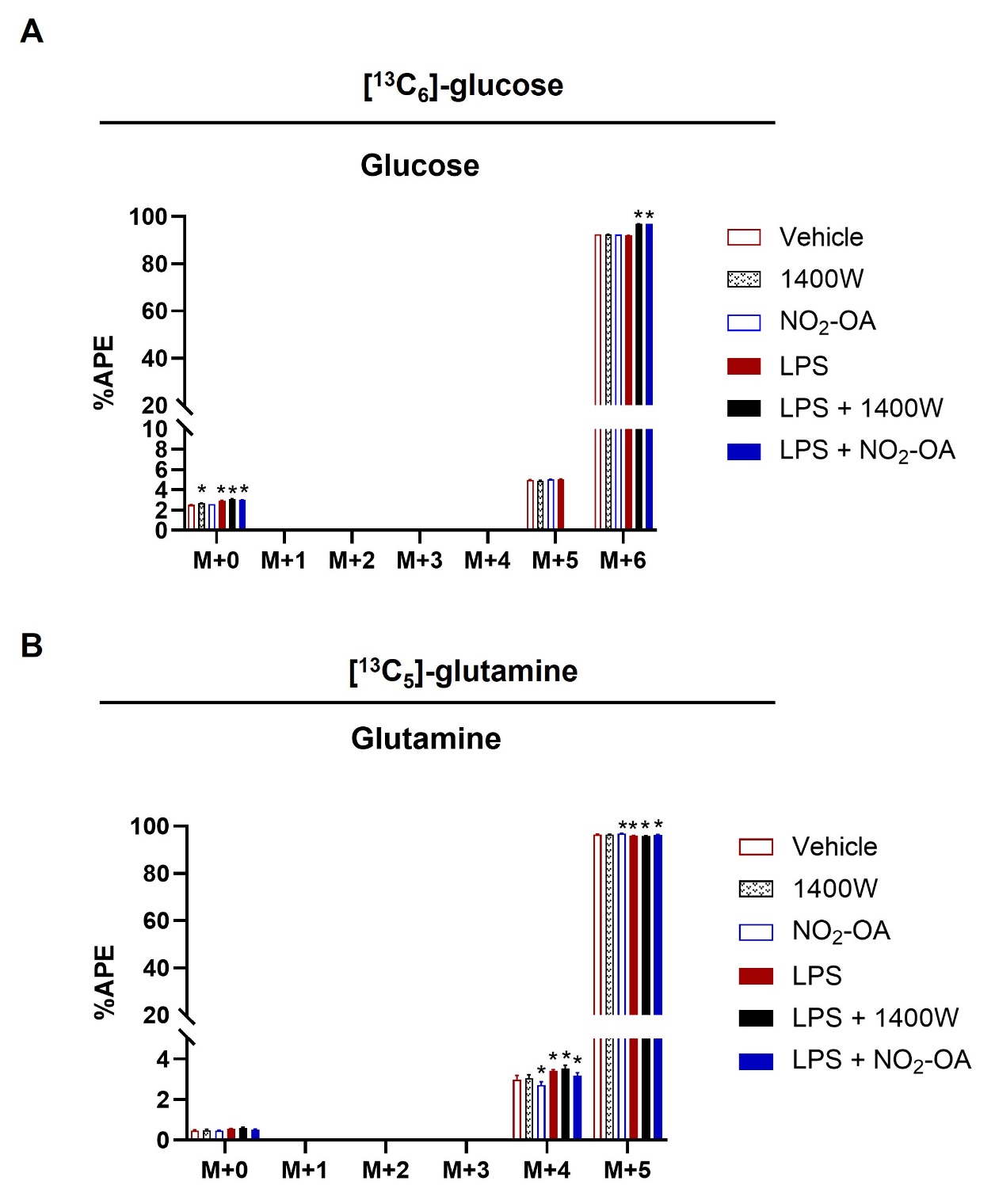
Supplemental Figure 4. Confirmation of ^13^C enrichment of glucose and glutamine substrates in RAW 264.7 macrophages.** Atomic percent enrichment (APE) was calculated for (**A**) ^13^C glucose ([^13^C_6_]H_12_O_6_) and (**B**) ^13^C-glutamine ([^13^C_5_]H_10_N_2_O_3_) under treatment conditions. p<0.05 when compared to control (*).

**
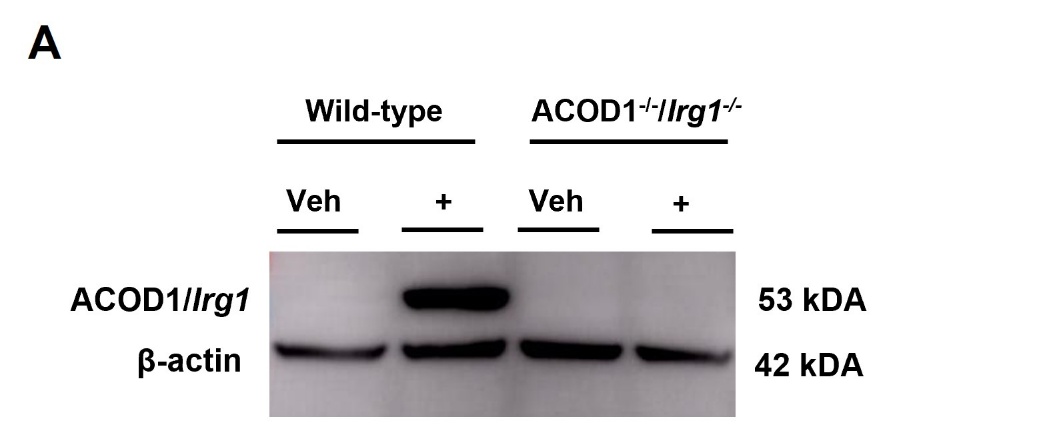
**

**Supplemental Figure 5. Confirmation of *Irg-/-* in RAW 264.7 macrophage**. WT RAW 264.7 macrophage and *Irg-/-* were activated with Klebsiella to demonstrate the knockdown efficiency of ACOD1 expression. *Klebsiella pneumoniae* (“+”) supernatant was prepared as previously described (51). Cells were incubated for 6 hr with 5% (v/v) *Klebsiella pneumoniae* supernatant as a positive control to confirm ACOD1 expression.

**
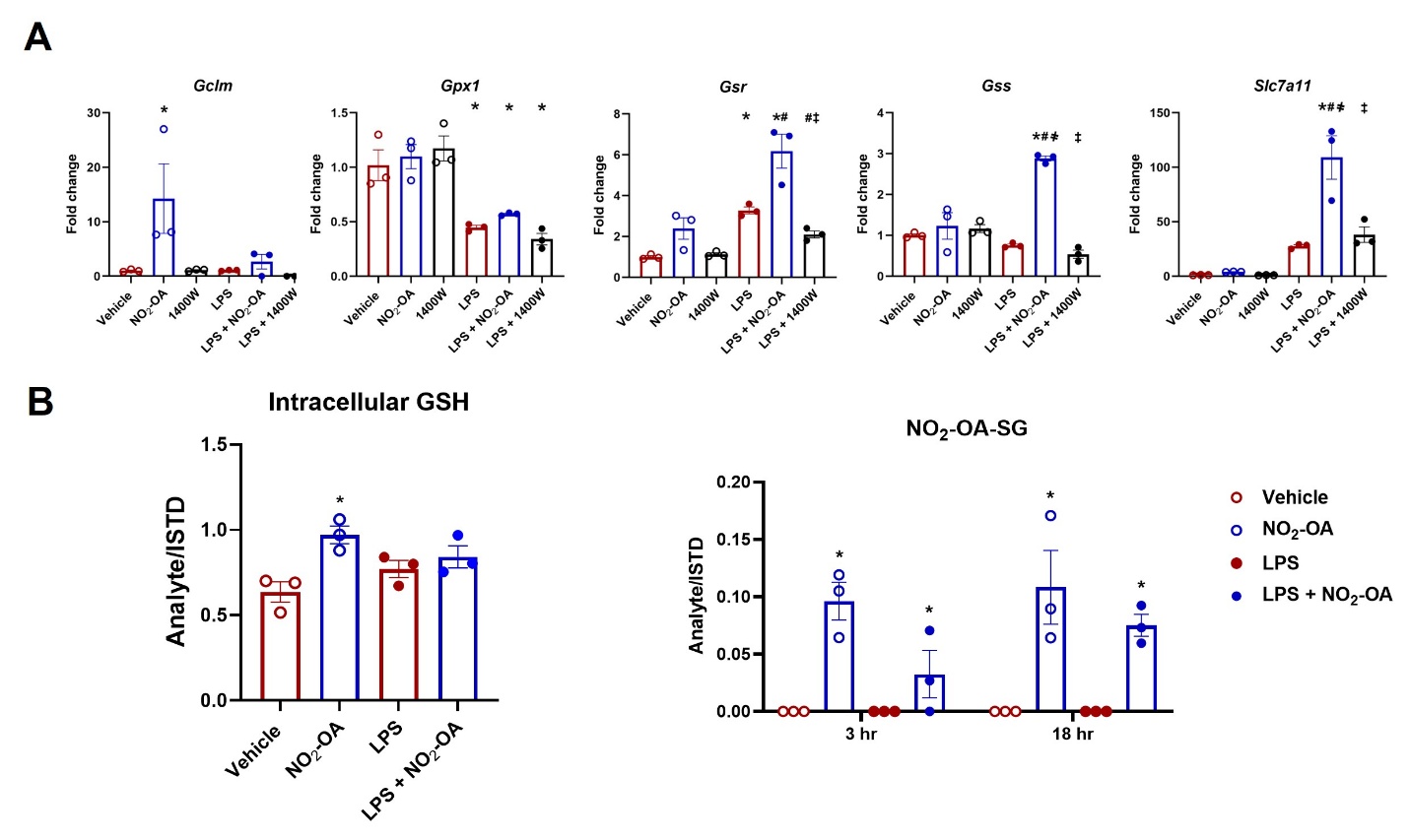
Supplemental Figure 6. NO_2_-OA repurposes extracellular glutamine for glutathione synthesis in BMDMs**. (**A**) BMDMs were treated with vehicle (0.01% DMSO, control), or NO_2_-OA (5 µM) with and without LPS activation (10 ng/mL) for 6 hr prior to collection in Trizol™ for RNA preservation. cDNA was prepared and PCR was performed using primers specific to modifier subunit (*Gclm*), glutathione peroxidase 1 (*Gpx1*), glutathione reductase (*Gsr*), glutamate-cysteine ligase, glutathione synthetase (*Gss*), and solute carrier family 7 (cationic amino acid transporter, y+ system) (*Slc7a11;* i.e. xCT). Data were normalized using GAPDH (control gene) expression and vehicle (control condition) and are reported as fold change (2^-∆∆CT). (**B**) Treated cells (3 hr) were collected in N-ethylmaleimide (NEM) derivatization buffer for quantification of GSH. (**C**) Media was harvested from treated cells (3 and 18 hr) and subjected to solid phase extraction to capture GS-OA-NO_2_ for quantification by LC-HRMS. Data points are technical replicates of BMDM pooled from n = 6 mice.

**
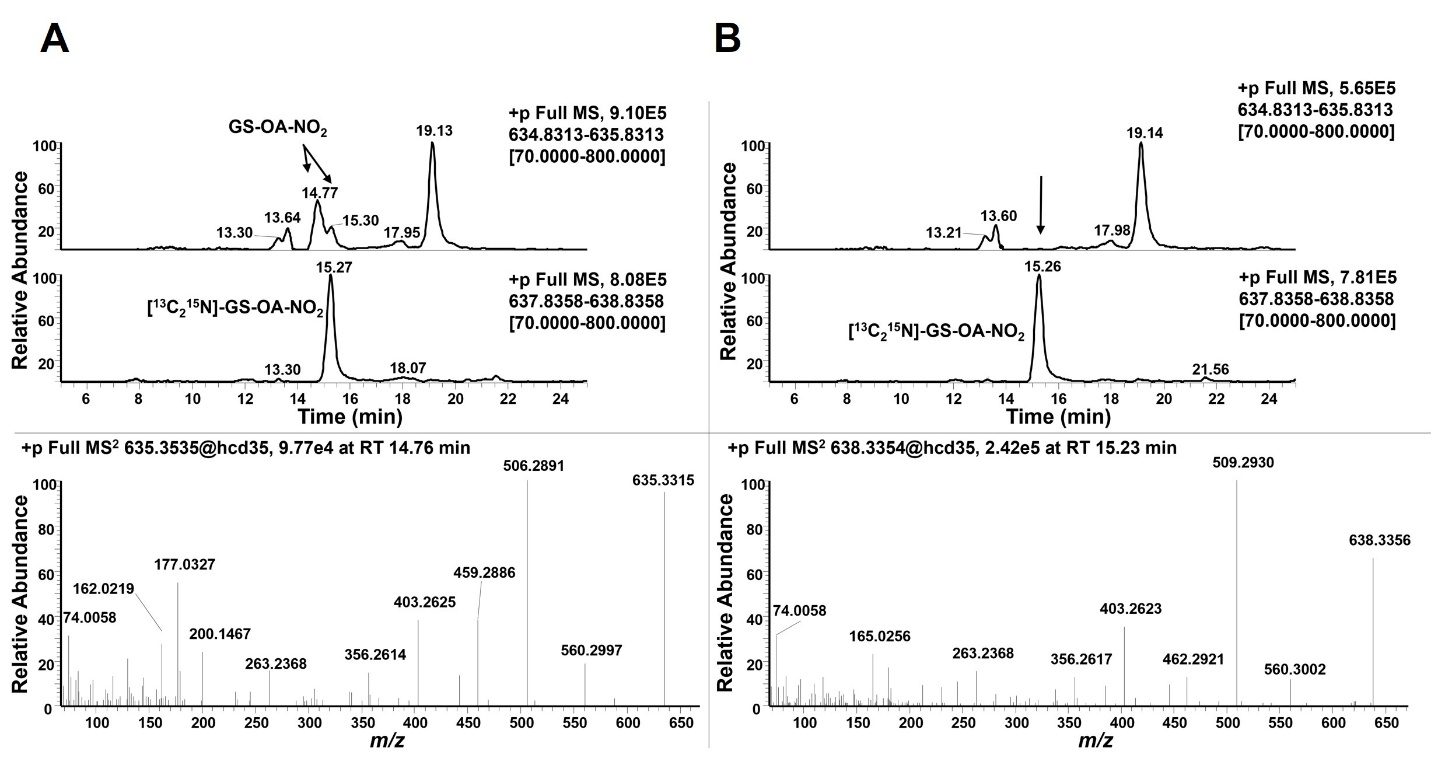
Supplemental Figure 7. Confirmation of GS-OA-NO_2_ formation in RAW 264.7 macrophage by LC-HRMS**. (**A**) The top panel shows the extracted ion chromatogram for GS-OA-NO_2_ that formed after treatment with 5 µM NO_2_-OA. The stable isotope labeled internal standard [^13^C_2_^15^N]-GS-OA-NO_2_ used to confirm peak retention time. The lower panel shows the product ion spectra for GS-OA-NO_2_ (*m/z* 635.3315) that was measured from RAW 264.7 macrophage supernatant. (**B**) Cells treated with LPS do not form the GS-OA-NO_2_ conjugate (top panel). The bottom panel shows the product ion spectra for the stable isotope labeled internal standard (*m/z* 638.3356), which is 3 amu larger ([^13^C_2_^15^N]-GS-OA-NO_2_) than the endogenously formed GS-OA-NO_2_. Corresponding diagnostic ion pairs include *m/z* 638.3356/635.3315, *m/z* 509.2930/506.2891 (neutral loss of 129 amu from GSH), *m/z* 462.2921/459.2886. and *m/z* 165.0256/162.0219.
